# Southern Dental Association

**Published:** 1885-06

**Authors:** 


### THE DENTAL REGISTER.

VOL. XXXIX.] JUNE, 1885. [No. 6.

### PAPERS, DISCUSSIONS AND PROCEEDINGS

REPORTED FOR THE DENTAL REGISTER BY MRS. M. W. J.

### Southern Dental Association—Seventeenth Annual Session—New Orleans, La.

FIRST DAY—MORNING SESSION.

The Southern Dental Association convened, in its Seventeenth Annual Session, at Tulane Hall, Dryades st., New Orleans, La., on Tuesday, the 31st of March, 1885.

OFFICERS.

President, A. O. Rawls, Lexington, Ky.

First Vice-President, W. R. Clifton, Waco, Texas.

Second Vice-President, T. S. Waters, Baltimore, Md.

Third Vice-President, B. H. Catching, Atlanta, Ga.

Recording Secretary, R. A. Holliday, Atlanta, Ga.

Treasurer, H. A. Lowrance, Athens, Ga.

Committee of Arrangements—J. R. Walker, J. A. Thurber, Jas. S. Knapp, of New Orleans.

The membership of the Association was well represented from nearly all parts of the country, among whom may be named Drs. McKellops, Spalding, and Morrison, of St. Louis; Cushing and Harlan, of Chicago; H. A. Smith and J. Taft, of Cincinnati; Bonwill, of Philadelphia; Patrick, of Illinois; E. Parmley Brown, of New York, and many others; also members of the National Association of Dental Examiners, and representatives from the State Boards of Indiana, Illinois, Michigan, Ohio, Georgia, Maryland, Mississippi, and Louisiana.

The Association was called to order at 10 A.M., President Rawls in the chair.

At this point Dr. L. A. Thurber delivered the following

### ADDRESS OF WELCOME.

MR. PRESIDENT AND GENTLEMEN.—By request of the Committee of Arrangements, I have the distinguished honor of welcoming you to New Orleans—to the same hall where fifteen years ago was held a meeting of the Southern Dental Association of the most gratifying character. Year after year we have seen this body steadily working to advance the destinies of a profession which honors every one of its members. To crown its efforts in the path of progress, this session is held in this Crescent City, chosen at this time by the civilized world as the grand exchange and point of display of all that can improve and benefit the condition of man. As dentists, as men proud of our calling, we have a right to a place in this Congress of human ingenuity; it is appropriate that we should meet here and show the world the accomplishments of an art founded on the highest principles governing science, and made famous by American brains and American hands. Ignored by the learned professions less than forty years ago, we to-day stand on a footing of equality with any craft whose mission is the alleviation of human suffering. And more than this, we are a recognized, independent, liberal profession. Our meetings are always fruitful of rich results. The more advanced tender freely the knowledge they possess, the experience they have gained, to the more humble or less privileged, with a generosity and kindness characteristic of members of one family. To a gathering of such loyal men I am here to say WELCOME! Welcome, with the hope that the deliberations held here will add one more stone to that monument which you are erecting to our profession, and which is consigned to immortality.

And now, my friends, you who have assigned to me this honorable task, if I have not welcomed your visitors in language more eloquent, and thoughts of greater depth, I indulge the hope that you will forgive my shortcomings, and only remember the sincerity of my purpose.

Dr. W. H. Morgan delivered a response on behalf of the Association.

The President then delivered his annual address, which included an able paper on the subject of Pyorrhea Alveolaris.

Discussion of this paper was deferred until the subject of pathology and therapeutics should be reached in the regular order of business.

Report of Executive Committee called for.

Dr. Walker, the chairman, announced the following programme for sessions, subject to the approval of the Association:

Tuesday, March 31, three sessions, 10 A.M., 1 P.M., 7 P.M.

Wednesday, April 1, the same.

Thursday, April 2, Dentist's Day at the World's Exposition; programme to be issued later.

Friday, April 3, clinics all day, in Tulane Hall.

Saturday, April 4, three sessions, as on the first two days.

After various suggestions, on the part of Drs. Catching, Taft, Wright, Thurber, and others, it was decided to adjourn from one session to the next, guided by developments; the programme for Thursday and Friday being accepted as announced.

The President stated that the Committee on Applications for Membership was in session, ready to give all needed information as to conditions, dues, etc.

Also that by the law of the Assiociation all dues must be paid before the privilege of the floor was allowed.

On motion of Dr. Chisholm, visiting members of the profession and all physicians were invited to participate in the discussions.

The hour for adjournment having passed, adjourned to meet at 2 P.M.

### FIRST DAY—EVENING SESSION.

President Rawls in the chair.

Dr. J. R. Walker being the only member of the Executive Committee in attendance, Drs. Wardlaw, of Augusta, and Chisholm, of Tuscaloosa, were appointed by the chair to fill the vacancies.

Report of Committee on "Dental Education" called for. Dr. W. H. Morgan, chairman, not prepared. No papers offered; subject passed.

Drs. O. Salomon and E. J. DeHart presented their credentials as delegates to the Association from the Louisiana State Dental Society, which were received and their names presented to the Committee on Membership.

The committee reported as having been passed upon favorably, the names of Drs. B. S. Byrnes, Memphis, Tenn.; J. D. Miles and J. B. Askew, of Vicksburg, Miss.; R. J. Miller, Jackson, Miss., and O. Salomon and E. J. DeHart, New Orleans.

On vote by ballot they were declared unanimously elected, and their names ordered entered on the roll of Members.

Report of the Committee on Hygiene called for. Dr. W. C. Wardlaw, chairman of the committee, read a paper, of which the following is a synopsis:

He spoke of the continual struggle between health and disease, physical perfection being an ideal aim, as perishable organs can not be made independent of laws. Efforts made in this direction have resulted in the formulation of general principles known as the laws of health, or the science of hygiene. The laws pertaining to the hygiene of the teeth take a wide range; they go back prior to the marriage state, in the choice of mates; but national laws on this subject will always be a dead letter, because men are governed by their passions. Spartan laws aimed at the "survival of the fittest." The medical science of to-day, on the contrary pursues and perpetuates the most undesirable progenitors. The race of horses can be brought up to a given standard by cross-breeding, etc., but men are not horses and cannot be made subject to such laws. The generality of mothers will not be guided in this matter; the requirements of custom and of fashion are more potent. We must look for practical results in the present generation. Men will say with Mark Twain: "Blame posterity: What has it done for us?" We can only give mothers the general principles that "cleanliness is next to godliness;" that clean teeth will not decay; that the teeth must be kept free from acids and from decaying, fermenting matters. Impress these ideas upon a mother with her first babe in her lap, and you lay the foundation of all hygienic law. She is not compelled to acquiesce, but you hold out practical inducements. With the rubbing of the first tooth with a soft rag, a habit is formed which becomes easy; the brush becomes as necessary to comfort as to health. The teeth are protected from the enemy. Appeals to her pride and self-respect effect more than all your prescriptions of medicine and diet. You can give oatmeal, brown bread, lacto-phosphates, etc., for their tonic effect; but do not believe that lime-water or inorganic elements will be assimilated; must pass through vegetable organisms. Ordinary food furnishes all necessary elements, but not as prophylactics. Every surface of every tooth must be cleaned thoroughly and regularly with a moderately stiff brush—horizontal motion is not effectual; few know how to reach the inner surface of the lower incisors. To cleanse the interstices use the quill tooth-pick, or silk thread for ladies; the hotel wooden pick is useless. Too persistent use or misuse of tooth-pick is one cause of Rigg's disease. Use ant-acid powders, but they must not be gritty. Objects to pastes and soaps as not soluble, and remaining too long in contact with the gum; may be pleasant in the mouth, but do not remove deposits. Exercise on good food is essential to the health of the teeth and development of the jaws. Soft boiled food bad. Chewing gum beneficial in exercising and cleansing. Tobacco-chewing retards caries and prevents deposits of tartar.

Dr. Wardlaw then read a paper from Dr. B. F. Arrington on the same topic.

Dr. Arrington took the ground that we needed common sense —practlcal suggestions, rather than theories. That the field was not so broad as generally supposed. When we went beyond the treatment of the teeth and disease of the tissues of the mouth, we were at sea--in deep waters beyond our depth, beyond our specialty. It is our business to preserve the teeth and gums in condition for comfort and usefulness. This is the limit of dental practice. If this is well done, patients will be satisfied. Considers treating and dieting with a view to future generations absurd and impracticable. Accept the situation, and our duty is plain. The teeth from the age of nine or ten should be examined frequently. The treatment of young teeth should be temporizing, not attempting to do too much; examining frequently and never waiting till decay is far advanced. The age of patient, locality, and structure of tooth must decide the material to be used. The removal of tartar will be appreciated.

Uses sulphuric acid for pyorrhea alveolaris, which it is as well to call by its common name—scurvy. Let the treatment be mild or heroic, as indicated. Would treat for months, if necessary, by local applications. Considered prescribing mountain air, sea air, etc., as absurd, and involving useless expense, as far as benefit to teeth went.

These papers elicited a lengthy and spirited discussion.

Dr. Catching said there were ideas in both papers that should be impressed both on the profession and on the people—should condemn all secret preparations, no matter by whom compounded, unless the formula was furnished.

The Tablets of Dr. J. L. Lyon are *probably* a good thing, as they have been on the market a long time, but we have no right to prescribe them so long as we do not know the ingredients. We want friction to cleanse the teeth, but we get no friction with soap, therefore useless. Would never use a wooden tooth pick again.

Dr. J. R. Walker said he objects to recommendation of *moderately* stiff tooth-brush. If 100 represents the range of tooth-brushes would never use more than 25. A stiff brush tears the gums and fails to enter the spaces between the teeth. The stiff brush is a prominent factor in the causation of pyorrhea alveolaris. Would use a soft brush, and soap too, after oily food, and brush from the gum to the cutting edge. Wooden tooth picks are an abomination and a snare.

*Dr. C. W. Spalding*—The subject of Dental Hygiene is an extremely important one. The teeth of children are too often neglected; sufficient attention is not paid to their cleanliness. The general impression among the people is that as they were soon to be replaced by others, they were of very little importance.

Had labored strenuously to correct these false ideas, but the means of reaching the masses are limited. There is a variety of opinion as to modes of cleansing. The gums require friction as well as the teeth. A stiff brush of good quality can be rendered flexible, but a common stiff brush will never be a good one. The manufacture of tooth-brushes needs remodeling as well as the habits of the people. The motion of brushing should begin on the gums and move towards the ends of the teeth. A stiff brush used across the teeth often does harm, but if used in the way I have indicated no injury will be done. If the brush handle is straight a rotary motion on the axis of the handle is an effective one. No dentist is warranted in prescribing secret preparations of any kind. He is entitled in advance to know the formula.

A dentrifice is chiefly useful in increasing the friction of the brush, soaps are lubricants and diminish friction. Tooth-washes are as valueless as water. Therapeutically uses local applications only for specific purposes, such as pyorrhea alveolaris and the devitalization of pulps.

True hygiene must commence in early life. If proper habits are formed in childhood there will be little trouble in after life.

*Dr. W. H. Morgan*—Both papers good as to general principles. Have failed to secure all the benefits that Dr. Arrington claims from sulphuric acid. "Clean teeth will never decay?" Do not know what that means. Decay in many instances does not result from foreign substances, as in case of vitiated fluids. If the acid theory be true, the mouth may be very clean, and decay be rapid.

Must reach further back for causes. It was said that "Mothers must have pleasant surroundings to secure good teeth for progeny." In the hills of Tennessee the mouths of children amidst surroundings of filth and squallor and absolute want, present a most favorable contrast to the teeth of the children in New Orleans. Keep the mother in general good health, but no special condiments required. Nothing in that theory. Had swallowed it when first advanced, but had reached contrary opinion.

No article of diet but what has in it more lime salts than necessary to develop the osseous system. But very small weight of lime salts in a dry skeleton. Huxley says not more than two pounds.

In rice, which is the poorest material used, there is 9-10 of one per cent. If three pounds is eaten in a year, and it takes seven years to wear out and rebuild the skeleton, there will be one pound of bone material furnished from this poorest of all material. It has been claimed that the Mississippi Delta has no lime salts! Look at this great river. It takes up the lime salts from the day it passes through, from the limestone beds of the Tennessee, from Ohio and New York, by the Wabash from Illinois, by the Red River from Arkansas, lime is brought, and spread all over this country forming this illuvial soil. The cereals are intended to meet our wants. Dentrifices and washes scour and correct acids. The result of my investigation is that the most rapid decay is when the fluids are decidedly alkaline. Do not say that alkali dissolves tooth substance, but that some chemical action takes place which favors decay. Am most concerned in case of alkaline saliva. Ammonia, when exposed to atmospheric action, develops nitric acid; this is in its nascent state, and is found by tests—not in the saliva, but attacking the tooth bone. We need something to neutralize alkaline fluids. As regards stiff brushes—were formerly universally recommended, to the terrible destruction of teeth. If the brush is too stiff the gums are destroyed, and the necks of teeth so exposed, that fillings soon become necessary, and in time a ribbon of gold will follow the edges of the gums, as the result of stiff brushes across the teeth. I prefer a napkin for friction, but remove debris from between the teeth by other means. I condemn all temporizing work. To preserve teeth must put in a good filling. If a six-year molar, at seven cut out the fissures and fill with gold. If you don't know how to use gold, use something else. It is a great mistake not to cut out fissures. Will soon have to do your work over again if you temporize. No matter what the age of the patient, do the work perfectly. Soap is a great cleanser; am satisfied with it in my own mouth. It dissolves oily and other deposits that adhere, so that very little rubbing is required. When the gums need local treatment I use astringent escharotics.

They control where I have failed with everything else. I use nitrate of silver when its use is indicated.

*Dr. Jas. S. Knapp*—Agrees perfectly with Dr. Morgan as regards tooth-brushes. The gums are injured and the teeth grooved. We often find loss of substance on the outer surface that can be accounted for in no other way. It does not look possible, but a simple experiment will illustrate. Place a tooth in a vice, and rub across with a fine point of wood dipped in charcoal. A groove can soon be worn. The instructions given are too obvious to need mention.

*Dr. J. R. Walker*—Endorses all that Dr. Morgan has said about filling children's teeth, and filling them well, but would sometimes use other material than gold—not because the operator is necessarily ignorant of the use of gold, but because the soft texture of young teeth makes other material advisable. Would never *temporize*, or do imperfect work, for any reason. Does not agree on some other points. Some facts need pointing out. Was glad to hear Dr. Morgan admit the difference in the character of the teeth in Tennessee and in the Mississippi Delta. The prevalence of poor, soft teeth in the latter locality, is due to the absence of lime-salts in food and drink ; appreciated his eloquent portrayal of the great Mississippi river, but unfortunately it is not that river water, with all those lime-salts held in solution, that is used for drinking and cooking purposes, but *rainwater*, which was described by Dr. Stienberg, as the "sewage of the air," which is destitute of organic elements. A recent trip through Texas had served to confirm him in his belief that the carbonate of lime is beneficial to the teeth. In the mountains of of Western Texas, where the carbonate of lime abounds, the natives have magnificent teeth, in strong contrast to those of the people in the Mississippi Delta.

It is advocated that the mineral elements must go through a preparatory process ; that it must pass through the vegetable kingdom, but he is convinced that the aqueous carbonate of lime itself is of benefit to mothers. In Western Texas there is a tract of country where the water is so thoroughly impregnated with the *sulphate* of lime as to be popularly known as "gyp-water." Had some curiosity as to what effect that form of lime would have upon the teeth, and was gratified to find the teeth of the natives of very superior quality. No grain is raised in that part of Texas ; they are stock-raisers ; they use St. Louis flour ; but they drink "gyp-water," and they have magnificent teeth. Dental operations are required only by recently arrived immigrants.

The geologist knows from the mineral formations of a region what its flora and fauna will be. The character of the teeth can be predicted with equal certainty from the same data. The geological formations which decide the quality of the water also decide the character of the teeth. If the absence or presence of lime has nothing to do with this difference in the character of the teeth, in the localities described, would like to ask the cause?

*Dr. W. H. Morgan*—The poorest food has sufficient lime-salts to give each individual three pounds in a year—it will give him all he needs. Yesterday, took a trip through certain portions of this city, where the filth was simply horrible; the stench was intolerible; foul gasses were bubbling up through the surface-water in the gutters. That is what is the matter with the teeth in New Orleans.

*Dr. Walker*—Admits the condition of the city to be most lamentable, but attributable to the unprecedented rain-fall of the past winter; but with all these bad sanitary conditions, the atmosphere is so purified—the impurities so continually swept away by the gulf breezes that we have no typhoid or diphtheritic epidemics, as in other less favored localities. Was in a certain town in Texas where the sanitary conditions were equally bad, and where typhoid fever was consequently almost a certain epidemic; and yet the teeth were firm and sound.

*Dr. B. H. Teague*—Whatever may be the cause of decay—whether acids or bacteria, or deficient lime-salts—the average dentist is more concerned with its prevention.

The simplest tooth-powder is the best, and that is English prepared chalk. A dentist makes his own tooth-powder; he knows its components; he has a right to recommend it. He gets very busy; his stock gives out; he sends to the nearest depot and orders a supply for convenience. If the formula is not given, he has no right to recommend it to his patients. Has a druggist compound a tooth-powder of which he prescribes the formula, and sends his patients there.

One idea as to tooth-brushes that has not been advanced: the bristles should be quite long—much longer than as generally used—serrated and separated into two or three rows. The bristles quite long so as to carry the powder used in a sweeping manner. Short bristles only scratch. With the long bristles there is more friction, which is needed for both gums and teeth.

*Dr. C. W. Spalding*—As to the constituents of our food, recent careful analysis of the kernal of wheat shows that the interior portion is not entirely starch ; the old ideas on the subject are not correct. There is a net-work of gluten pervading the starch layers, and a corresponding distribution of lime-salts all through the grain of the wheat.

Every article of food contains enough tooth material, but we often do not get the benefit of that material ; because of defective assimilation. As to the administration of certain elements, for pre-natal effects, there is no question as to the benefit derived.

If a mother partakes of certain elements, the child is benefited by it. These are verified facts. The general condition prevailing in a family may be avoided in the children. But while the phosphates in the food are not assimilated, the phosphates as medicine are beneficial. *Why* is not understood. One fact in illustration : years ago, phosphate of lime was found beneficial in certain diseases among the English soldiers in India. The Home Government advertised for large supplies ; bids came from various sources ; one was less than half of any other ; this was accepted, subject to test ; and, on testing, was reported chemically pure ; this phosphate was purchased and shipped to India and proved to be absolutely useless. Why ? Investigation showed that it was the mineral phosphate. The lime phosphate that had been previously used was bone phosphate. It had been acted upon by animal organisms. We are not as a rule, able to assimilate mineral elements. They must first be acted upon by the organizing forces of the vegetable kingdom. Bone phosphate is beneficial ; mineral phosphate is useless.

*Dr. J. Taft*—It may be well to inquire ; why it is that there is occasion for so much effort to keep the mouth pure and clean ; what are the sources of impurities? With a clear conception of the causes, we would better know how to prevent. Among the various sources may be named the accumulation of debris, which lodge and are retained upon the teeth ; the imperfect use of the teeth—insufficient friction from mastication. We use too much food not requiring mastication; soft food. Those habituated to soft food have pasty deposits, which are very often attributed to vitiated fluids, or deposition of salivary calculus. This can be avoided by the thorough mastication of proper food. The condition of the secretions has also much to do with the condition of the teeth. Another cause is found in the use of improper food, which often irritates the mucous membrane of the mouth and even of the alimentary canal. The saliva is also vitiated by undue stimulation of the salivary glands, as from the use of tobacco. If thus habitually stimulated, common food will fail to excite the proper action, and other fluids will be required to compensate. Saliva is also vitiated, and impurity and uncleanliness of the mouth occasioned from the habit of mouth-breathing, especially in sleep. The mouth becomes dry; the mucous is changed, becomes thick and agglutinated; the mouth is pasty if not dry, with an unpleasant taste. This is, in part at least, the cause of the unpleasant taste in the mouth when suffering from a cold, and one is forced to breathe through the mouth. In every instance when the teeth are thus foul, from any of these causes, they suffer. Shall we disregard all this, and resort simply to the tooth-brush and the tooth-pick? No, we must remedy these conditions. If the condition of the mouth was always what it should be, we would have very little use for the tooth-brush.

If we avoid all causes of impurities we will have no more use for the tooth-brush than other animals. Have known persons who never used a tooth-brush, but whose mouth and teeth were clean and pure. Let every person masticate well; use proper food; avoid all causes of abnormal secretions; avoid all irritants of the mucous membrane, and there will be much less use for dentrifices and tooth brushes. Respiration should be entirely through the nostrils. This habit should be carefully cultivated, and every effort made to overcome the opposite habit. Every person in good health will have a pure sweet breath if the mouth is habitually closed in breathing, and especially in sleep. Education on these points devolves upon the dentist.

*Dr. A. O. Rawls*—Will Dr. Spalding please give the reason for the bad taste in the mouth referred to.

*Dr. Spalding*—It results from the evaporation of the watery portion of the saliva, which also undergoes rapid chemical changes when exposed to atmospheric currents.

*Dr. W. H. Morgan*—Any person can readily experiment for himself by voluntarily keeping the mouth open—say while reading—for one hour. He will find his mouth foul and sour.

*Dr. Spaulding*—Fermentation will as surely take place in the open mouth after eating food containing sugar, as in the juice of the grape when exposed to the air. Dr. Taft spoke of irritation of the salivary glands as affected by stimulation. The saliva is alkaline; but mixed saliva when the mucous is in excess, becomes acid. The salivary glands are very deeply imbedded, and are not readily stimulated by excitation of the mucous membrane except as a reflex act.

*Dr. Taft*—Said that he referred to the salivary glands, as they respond promply to every undue stimulus, but agrees also to the vitiation being due in part to the admixture of vitiated mucus secretions. We should do all that we can to educate the people in right habits, rather than have to combat disease. Had used the phosphates in mineral form, for many years, as he believed with good results. Could site many cases. Would mention one family where the father came from New England, the mother from New Jersey, both having fine strong teeth. They moved to a sandstone country, where there was little or no lime in the vicinity. Five children were born, and all had very defective teeth, and and lost them early. In many other families in the same vicinity the case as to the children was the same. How much this was cause and effect, we can only judge. The water of that locality was perfectly free from lime: what is called "soft water."

*Dr. W. N. Morrison*—Would say one word, with regard to to ordinary food containing sufficient tooth-elements. This may be true, but in the early age of children, not enough of this proper food is taken. The desire for cereal foods, wheat, rice, barley, or a meat diet, is diminished by the use of *sweets* between meals. They don't get enough of the proper kind of food to supply the elements of tooth substance because they have no appetite for good, plain food. Their stomachs are filled, and the secretions exhausted, between meals, and they refuse good plain food at the table, because they have no appetite for it. This point should be borne in mind in instructing patients. With regard to the prevention of decay, one promoting cause is the long intervals customary between meals. Decay progresses in this interval. If we ate like the animals—small quantities at frequent intervals, never overloading the stomach, allowing no interval for this work of disintegration, we would have better teeth. As to brushes, I approve of a very nice fresh brush, with bristles of medium length and serrated, but ordinary brushes are made hind-side-before. The strongest and best part of the brush should be at the end, to endure the wear of friction. For cleansing spaces recommend narrow strips of rubber dam, instead of silk-floss; always dismiss a patient with a supply.

*Dr. J. S. Knapp*—Does not agree with Dr. Morrison as regards frequency of eating. Believes that by eating too often, the appetite is destroyed. Saccharine food is very bad. Plain substantial food at stated intervals.

*Dr. J. R. Patrick*—Has listened to the discussion with great interest. It is undoubtedly true that the attempt to convert internal membrane into external membrane, by keeping the mouth open, is the sum total of the whole trouble. Internal cannot be converted into external membrane, without creating unhealthy tissues. There is no question but that man has advanced from a lower to a higher type as he progresses in the march of civilization. Where vegetation exists, animal life can be sustained. It is not necessary to have limestone in order to have lime. Every blade of grass, every tree, every flower, is at work transmuting the inorganic elements to the organic state, and there the animal creation finds it, though they never saw Graham or cracked a grain of wheat. You cannot send your elements direct to the knee-joint, or the big toe, or to one particular bad tooth. Can you say to the phosphate *go* and have it go direct to the right place? Can you turn the gas on, light one burner and skip the next? The elements are appropriated in a general way; we try to do too much. We hold out too many inducements that will never be fulfilled in reconstructing teeth. Animal tissues are thrown out; organic elements are deposited. The forming process of teeth is from centre to circumference; the lime salts are thrown out; the teeth are centripetal in growth, bones are the reverse. In old age when a tooth has served its purpose, it has no internal cavity, in its normal condition. There is a filling up, to a certain age, in a permanent tooth; there is a retrograde, physiological process. I am satisfied that when a patient is excreting certain elements, it is not necessary to give more because they don't appropriate what they have already. A tooth is incapable of repairs except in broken cementum, which is similar to bone. Are the teeth of pregnant women broken down to supply the fœtus? I don't believe they are; I can't see it possible. Women take more care of their teeth; have greater pride in their appearance; visit the dentist more. I should want very positive proof on that breaking down process. A child excretes no phosphate of lime or of magnesium when making teeth, as in later life, nor does a pregnant woman.

*Dr. Taft*—Is not prepared to endorse the idea that the teeth cannot be nourished by the administration of certain elements, without supplying all other points with the same material. A certain element goes where it is most in demand. If a bone is broken the proper elements go there to repair. Lime does not go only to the muscles; it goes to the bones and teeth where needed. We do not need to *send* it; we only have to furnish it, and it will go to the right place, but it cannot go if not furnished. Where the teeth are poor, the fontanelles are wide open; there is a general deficiency. With prompt dentition there is prompt closure of the fontanelles. With poor teeth, the bones are soft; sometimes so much so as to bend without braking. If the proper elements are administered, they will go where they are most needed.

*Dr. W. H. Morgan*—Dr. Patrick is a little misty in his physiology, and I do not agree with his osteology. I think the bones are formed like the teeth; it is the same process. This is the view of all comparative anatomists in the world. When the nerves of taste detect a flavor, you have increased flow of saliva—

a reflex process. As to Dr. Morrison's idea of not eating often enough; if there is nothing to promote a flow of saliva, too much mucus will be secreted.

*Dr. Spalding*—Only one word more on assimilation. We know that the blood contains all the elements, and that certain proportions are distributed to different organs. All organs do not take equal supplies of the same element. Assimilation depends more on the condition of the tissues than on the supply of elements. The heart sends it to the door, but if the door does not open, it will not be received.

*Dr. J. R. Patrick*—It is lack of function, and it is useless to send additional supplies.

*Dr. Spalding*—The elements are sent out uniformly. Blood plasma contains all the elements. Here are the elements: take what you require. Assimilation depends on the condition of the tissues; not on the blood plasma. Our food contains all the requisite elements; the blood carries them all. Both of the gentlemen are right, but they both stop short of the main point. A flow of blood to the organs is required to stimulate it to assimilation. The teeth require exercise to excite the flow of blood to stimulate assimilation. Hard coarse food creates this condition. The muscle of the blacksmith illustrates the idea. If the arm is tied up it will waste away. The teeth require exercise on proper food.

*Dr. Robinson*, of Michigan—We don't know as much as we think we know about the mysteries of life. Life is a unit, flowing from centre to circumference. God is the great centre of the universe, and all nature flows from God the centre.

*Dr. J. R. Walker*—One word in reply to Dr. Patrick. We need to supply the elements which are excreted. I do not endorse the chewing of tobacco or gum to excite flow of saliva. As to the point at issue between Drs. Morrison and Knapp, I think we must "split the difference." Have our eating hours neither too frequent nor too far apart. Allow time for digestion, but not for undue secretion of gastric fluids.

Subject passed.

*Dr. H. J. McKellop*—What are the arrangements with regard to the exhibiting of appliances? As chairman of that committee I have induced a number of gentlemen to come here, some from a considerable distance, bringing a number of valuable and interesting appliances and improvements, but I have not learned what the arrangements are.

*Dr. J. R. Walker*—Chairman of the Executive Committee stated that Friday had been set apart for clinics and the exhibition of appliances.

The application of J. Rolla Knapp for membership was announced as having been passed upon favorably by the committee.

A vote was taken and he was declared elected, and his name ordered placed on the roll.

Adjourned to meet at 9 A. M., Wednesday, April 1st.

WEDNESDAY, APRIL 1.—SECOND DAY—MORNING SESSION.

Called to order by Dr. B. H. Catching, third Vice-President.

Minutes of yesterday's sessions read and approved.

Dr. H. J. McKellops moved that half an hour, after the adjournment of the morning session each day, be set apart for the exhibition of appliances and improvements.

Seconded by Dr. W. H. Morgan, and carried.

Dr. John W. Adams, of New Orleans, was recommended by the committee, and elected to membership.

Report of the Committee on Pathology and Therapeutics called for.

Dr. Wm. H. Atkinson, chairman, not present. No papers presented; subject passed.

Report of the Committee on Histology and Microscopy called for. Prof. J. Taft, chairman; no report ready; no papers presented; subject passed.

Report of the Committee on Chemistry called for. Dr. J. S. Cassidy, chairman, not present; no papers presented.

The death of Dr. S. M. Prothro, of this committee, announced.

Committee appointed by the chair to draft suitable memorial resolutions.

Report of committee on Operative Dentistry called for. Dr.

J. C. Ross, chairman, not present. No papers offered by members of the committee.

Dr. Wm. N. Morrison, of St. Louis, read a paper, of which the following is a brief synopsis:

His subject was the too frequent "reckless sacrifice" of tooth substance in opening up the pulp-chamber and root-canals of dead teeth; the free cutting and removal of solid dentine for convenience of operating in filling root-canals in teeth that require the most strength, because they are dead teeth. Burring out an aperture one-eighth of an inch in diameter, drilling out the nerve canals, etc., leaving only a thin shell of enamel; such teeth readily breaking down under ordinary use in mastication. Dr. Morrison took the ground that there was no excuse for such wholesale destruction of valuable tooth-material. On the contrary, would save all the crown-substance of dead teeth. The range of possibilities was very great. For filling root-canals would use only very soft, pure gold wire, the exact size of the natural canal, never drilling or trimming the latter. Would get the exact size required by repeated trials, using oxy-phosphate of zinc, or gutta percha to fill the interstices. The wire used being very soft, would take the exact form of the root-canal—almost an impression. Introduces the oxy-phosphate with a tampon of cotton, using bibulous paper to absorb superfluous fluid. Claimed that any ordinarily skilled operator could remove the contents of canals and pulp-chamber through an opening in the center of the crown of 1-16 inch in diameter. No opening needed more than sufficient to allow a broach to reach the canals.

Also deprecated the system of over-treating. Described at length a case in which he had filled a large mesial cavity in a tooth where the pulp was in doubtful condition. Being obliged to devitalize the pulp later on, he did not remove the mesial filling, but drilled a 1-16 inch circular opening through the center of the crown. Devitalized with arsenic. In removing tissues found a nodule of secondary dentine the size of quail shot. With a barbed broach he tore the tissues from the nodule, the latter falling back when it reached the aperature through which it could not pass. While filling one root, he rolled the nodule over the opening of the other, finally impacting it in the filling of the pulp-chamber. In all cases he adapted the instrument to the cavity, rather than cut away the tooth to suit bungling instruments.

A spirited discussion followed the reading of Dr. Morrison's paper.

*Dr. J. S. Knapp*—While it is undoubtedly true that a large amount of a valuable tooth material is sacrificed for the convenience of the operator, the position taken by Dr. Morrison touches the other extreme. The aperture he describes, through which to remove the contents of root-canals and pulp-chamber, was too small for convenience. In the case described he would not have opened through the sound crown, when there was a filling in the tooth already. Could have reached the nerve canals more readily from that direction. In all cases would make a larger opening for convenience of operating and seeing.

*Dr. W. H. Morgan*—Had listened with interest to the excellent paper. The general idea of preserving tooth-structure is correct. In proportion as you cut away dead tooth you lessen its time of usefulness, because dead tooth will in time disintegrate. Round opening is more difficult to work through than a slot however narrow.

Considered his next step radically wrong. The worst condition and the worst time for filling was immediately after removal of contents of dead tooth. The semi-serous contents of the tubuli would putrify, if not disinfected. *Must* give antiseptic treatment before filling, to prevent mephitic gasses and periostial troubles.

*Dr. J. S. Knapp*—Would ask Dr. Morgan what is left, after the pulp tissues have been removed?

*Dr. Morgan*—The semi-fluid contents of the tubuli, which will putrify. I cut out the root-canals and pulp-chamber freely, and remove all the dentine, in which the contents of the tubuli are liable to decompose. Fills the roots in much the same way as Dr. Morrison, but has screw-threads on the wire. Removed a tooth recently in which there was a ball of ox-chloride. beyond the foramen. Was doubtful about extracting, but glad when I saw the condition, though nature might have expelled it. Would not cut away unnecessarily; had no pride in a large filling, as such.

*Dr. Parmley Brown*—Dr. Morrison's paper has waked us up. He is old enough and has had practical experience enough to know what he is talking about, but I don't advise every one to try his method. He might succeed and again he might not. I disagree with Dr. Morgan as to the use of oxy-chloride Prefer gutta-percha.

Dr. Morrison's paper should be copied into all the journals. Would wager for his success in filling *immediately*.

Ten years ago had a patient from Charleston. Pulps of ten superior anterior teeth had been devitalized six months previous, at her own request, as she told me to prevent suffering while being filled.

The fillings were all loose; springs of puss exuding. Examined well, and decided to put the dam on and fill them all before she left the chair.

I removed all the dead pulps, burring only sufficiently to remove the fillings. Filled immediately with gutta-percha, and sent her away with warning of possible swelled face. She had no trouble, and ten years afterwards the gums were quite healthy. Dr. Morrison is right in working through an aperture as small as he can, and I am right in having it as large as I require. I would not remove a permanent filling if another opening would suit me as well or better.

*Dr. W. H. Morgan*—Dr. Brown misunderstood me. I propose to remove all decomposed matter. He succeeded in this case because all matter had decomposed. The tubuli are in better condition when pulps are just removed to absorb antiseptics than when there is an old abscess.

(*Brown*—Mummies were not decomposed!)

*Morgan*—No, because antiseptics were not then used. In his case, the pulps and the tubuli had undergone all their decomposition, and one hour's treatment with antiseptics was all that was necessary.

But after the removal of fresh pulps, mephitic gasses will be formed, and give trouble. They will pass through the foramen and abscess will form. Abscess follows decomposition. If there is nothing left to decompose, you are safe. If everything is removed, you will have no abscess.

*Dr. Brown*—Where is the proof that the contents of the tubuli will die?

*Dr. Morgan*—Take a strumous, or scrofulous patient. Devitalize and fill immediately. In two months you will be satisfied; your nose will tell you. There will be decomposition in the dentine.

*Dr. Brown*—I never made the test, and never expect to have a chance. I succeed every time. The filling goes in immediately after the nerve comes out.

Drs. Morgan and Brown called to order, as the law of the association prohibits more than one speech, and ten minutes to each member, until all have been heard.

By unanimous consent, they were allowed to continue *the dialogue.*

*Dr. Morgan*—I don't understand Dr. Brown. If he has never had an abscess, he is the only man living who can say as much. If there is an abscess within a year, five cases out of ten will give trouble in time. It may not be in five but it will come.

*Dr. Brown*—I did not say I *never* had an abscess. In the case of the lady described, I sent her home, anticipating trouble, but there was none.

*Dr. C. W. Spalding*—Both of these gentlemen are right, and both are wrong. It all depends on the individual case. In some cases it will do, in others it will not; as in the teeth of young children for instance, and those of scrofulous subjects. I endorse Dr. Morrison's views, with some modifications. I have also found teeth in exactly the condition described by Dr. Morgan hundreds and hundreds of times. "Shall we fill immediately?" is the question. As a rule I remove the soft tissues, treat with antiseptics if required, and fill immediately. As an antiseptic I use mercuric bi-chloride 1 to 1000 in distilled water. If water is not pure, the bi-chloride may be decomposed and thus become nugatory. All local medication except for purificatory purposes is unnecessary and often injurious. If the tooth is not so sore as to give pain on pressure, I proceed with the coperation without regard to condition outside the root. Inflammation of the peridental membrane is due to extension from the pulp, or it proceeds from the effect of irritating gasses generated within the pulp chamber or canal. If these are rendered pure, and air and fluids of the mouth excluded, you are following nature's methods, and will be successful. Remove irritating causes and administer proper prophylactics and abcesses become almost unknown. In medicine I am a homeophathic, as some of you know, and the prophylactics are prescribed in accordance with that system.

One word as to Dr. Morrison's method of root-filling. Filling roots is an uncertain operation, and we should adopt that method which gives the most positive results. If oxy-chloride of zinc or liquid gutta-percha is pumped into the canals we do not know whether the canals are completely filled or not. If the canals are not well dried the uncertainty is still greater. The gold wire or gold strip method is a far more positive one. You can gauge the length of the root with tolerable certainty and can almost know whether you stop the foramen or not. I used gold wire for this purpose more than thirty years ago, but Dr. Morrison had improved upon the old idea. I believe I have tried about all the modes of filling these canals, except that abomination, the cotton and creasote method, and I feel sure the gold wire method in connection with gutta-percha is decidedly the most certain. After the wire is satisfactorily fitted into the canal, mark at the open end of the canal, withdraw it and pump in the gutta-percha, or oxy-chlorine, and immediately re-introduce the gold and the mark shows if it has been properly carried home. Cut or break it off and let it remain. To my mind this is the nearest approximation to a positive result of any mode with which I am familiar.

*Dr. A. W. Harlan*—I was not here to hear the reading of Dr. Morrison's paper, but have listened to the discussion.

There are doubtless many young men here, who have not had thirty or forty years experience, but we are all willing to be instructed ; we are all desirous of doing the best possible. There has seemed to me to be a lack of definiteness.

Does all this refer to cases when the pulp is to be extracted? When is the pulp to be extracted after devitalizing? You wish to prevent abscess; you must be governed by well defined and scientific methods.

If the pulp is destroyed to-day, according to the laws governing physiology, changes from a physiological to a pathological condition must follow. When we produce an eschar, will nature remove it to-morrow?

Eight days will elapse before tissue destroyed by eschar will be removed. Is it not natural that the same law should apply to destroyed pulp? Remove at once? Remove while wet? Adjust the dam, and prevent mixed fluid from entering, the latter will produce putrefaction. The fluid contents of the tubuli must be removed, and everything hermetically sealed. It is not filled so thoroughly as to exclude air and water, we will have bad smelling dentine in every case. With the above precautions we will not have trouble even with wrong methods. The necessity for treating is an erroneous idea. All that is necessary is to extract the water. With carbolic acid? No. With creosote? Not at all. What then? I reply, with absolute alcohol. Alcohol will extract the water perfectly, and will not coagulate albumen.

As to the treatment of pulpless teeth, no gentleman could fill a pulpless tooth for me at once, if discharging from the canal, and no fistulous opening.

We have no right to inflict such suffering; the consequences may be very serious; may result in death. That all septic matter must be removed, no one will question. If the canal is filled under the above conditions, the alveolus must be bored before dismissing the patient.

The necessity of treating through the fistulous opening is not obvious. If the pulp chamber and canals are thoroughly cleaned, and filled immediately, there will be no necessity for treatment. Let some one who has tried treating for months, try this method; you will see your success. There is no necessity for wasting tooth-substance to reach the canals. I don't care what you do if you don't use cotton to fill the roots. Seal the apex, no matter what with—gold, wire, lead, gutta-percha, oxy-chloride. Wire alone cannot possibly make a perfect filling. Have repeatedly extracted teeth with curved roots, not filled to the apex, where wire was used, must fill with plastics which will reach the unequal surfaces.

The average practitioner is incapable of filling with wire with uniform success. And many do not know how to use plastic materials; either oxychloride and oxy-phosphate will harden before they get half way to the apex. Use gutta-percha; not the little cones which come for that purpose. Never heat gutta-percha in a flame or in a hot dish. Use it cold and drawn out to its smallest possible compass; sufficiently rigid to go to the apex. I will be happy to show the method.

*Dr. Williams* (Ky.)—I find good features in plain common sense. In a room where smallpox has been, the carpet and curtains are not left in position to be disinfected. It is not common sense to disinfect surplus material. Remove all surplus material, and then with simple means the room is easily and thoroughly disinfected. As with the room, so with a tooth. A gentleman (Dr. Spalding) spoke of the uselessness of treating two or three weeks. Seal the apex; remove the surplus; disinfect.

A gentleman spoke of taking the impression in gold wire. I use a lead point, like the broken-off point of a sharpened pencil. Chip off one-sixteenth of an inch. After washing out the canal, I insert this little point, and thus close the door. I then remove the carpet and curtains. The tubuli contain more than the canals. In every direction I remove the dentine. In a few days I can thoroughly disinfect. Then use oxy-chloride or gutta-percha. The door is closed; all is sweet and clean. The whole matter is contained in a nut shell.

*Dr. Richie* (Texas)—Dr. Brown said he has no time to spend in treating teeth, neither have his patients. The time is not far distant when capping nerves will be left to those who do not attend associations. We have no right to impose upon our patients. Capping nerves is the greatest of all impositions.

Beautiful crowns are constructed, but in a little while peridental inflammation sets in; suppuration follows; the fine crown must be removed. The result of capped nerves is dead teeth, discolored teeth, springs of pus, septic matter carried into the stomach, the breath tainted, the health injured, and neuralgia.

The patient does not come back to the dentist; he goes to the physician, and there is a big bill to pay. If the physician only knew it, this neuralgia is due to capped nerves. I am opposed to capping nerves. I strike at the root of the matter in the beginning. I take the nerve out surgically; use local obtunders; use carbolic acid to coagulate the contents of the canaliculi; keep foreign matters out; fill the nerve canals with cotton and antiseptics to absorb fluids. In a week I fill permanently. For the roots, have tried gutta-percha, but my instruments go through; liquid plastics go through the foramen; elements clog before they reach the apex; wire projects beyond. I cut away for a clear opening, and use Robinson's fibrous material; dissect it and get long and slender fibers; lubricate the canal with eucalyptus oil; then introduce one thread after another, until the apex is closed. I then disinfect and bleach, and complete the filling. Would do away with capping nerves as unfair to patients, causing severe suffering, and taking our time to do the same work twice over.

*Dr. Walker*—This discussion affords a fine illustration of the benefit of attending associations. The ground has been traveled over in all directions. Will not detail my methods, but will give one or two points. In using plastics for roots close the foramen with gold or lead or whatever the particular case may require; when gold or lead will not reach in your hands, use gutta-percha. Fill with judgment as well as with your other materials; be guided by the circumstances of each case, but close the foramen thoroughly; shooting around a corner with metals is beyond my reach, but gutta-percha will do it. I then take a broach wrapped with cotton to pack the oxy-chloride, the broach will bring away the cotton but leave the oxy-chloride. As regards devitalization, I am in favor of preserving the nutrient functions. Devitalize when necessary, but save the nerve as long as possible. Keep a capped nerve under your observation, under a temporary filling for several days. I make the temporary filling of artificial dentine. Hill's stopping or other form of gutta-percha, or any material easily cut down. If no disturbance ensues within a reasonable period, then cover with permanent filling. This is better than wholesale devitalization for fear of possible trouble.

*Dr. J. A. Robinson* (Mich.)—Much depends on the instrument with which to fill the canal. Take a spring broach, twice the size of a horsehair to carry material to the apex, draw the end across your oil-stone and blunt the end, and it will not go through your material. The canal must be thoroughly cleansed. The best, the common sense test is the odor. For thirty years I have insisted that no tooth should be extracted except with the fingers. I use arsenic occasionally, but I cap the nerve whenever my judgment dictates its use; we are often too timid. When oxychloride or oxyphosphate is to be used, mix with carbolic acid to the consistency of cream, flow over the cavity, then use it thicker and press hard. It will cause pain for a few minutes.

*Dr. Chisholm*—Have had some experience in capping nerves. I use the old wood creosote, or oil of cloves with oxyphosphate. Where the symptoms are favorable, the nerve will live ten or fifteen years. I have a thousand cases that are doing good service.

All these different views make the field broader; many valuable ideas have been advanced. Have cured many cases by one treatment. On the other hand have had cases that required treating for months, but with ultimate success. In one family of consumptive tendency, it will return every spring. Sometime since in cutting an apple I severed the tissues of my thumb, my wife applied spirits of turpentine, and there was no soreness. Just at that time, in excavating a large cavity for a young lady I cut the nerve. I promptly applied spirits of turpentine and sealed it up, and it gave no pain. Have since tried it several times with the same success. This will perhaps remind you of the medical student who seeing the hospital physician allow pork to a little girl ill of pneumonia, who recovered, wrote in his memorandum book, "pork good for pneumonia," but his first pneumonia patient for whom he prescribed pork died.

The hour for adjournment having arrived, the programme for Thursday, April 2, Dentist's Day at the World's Exposition, was announced.

Adjourned to meet at 2:30 P. M.

SECOND DAY, AFTERNOON SESSION.

Called to order by Dr. B. H. Catching, third Vice-President. The discussion on Operative Dentistry was continued.

*Dr. B. H. Teague* said that we often failed in treating pyorrhœa alveolaris, where there was necrosed condition of surrounding bones because of impure sulphuric acid. As to capping pulps, facts were better than theory. There are two points to be kept in mind. 1st. No pressure on the pulp. 2d. Fill with a non-conducting substance. In one case where he capped a nerve with Weston's nerve caps and filled with oxy-phosphates, within twenty-four hours he had hard work to hold his patient while he removed the filling. Within a few hours he filled again with the same material, first washing gently with carbolized water, and then flowing oxy-phosphate over the pulp, finishing with a good amalgam. The tooth was very tender on pressure, and did not respond to thermal changes, and had the trouble occurred a week or ten days after filling he would have concluded the nerve had died; but the nerve is alive to-day, responding to thermal changes. A newly capped nerve will not bear the pressure of mastication at first.

*Dr. Reese* said that the one important thing in filling roots was to know that no impurities are left—we have a sure test—and that is permanganate of potash. If it does not turn brown in the cavity there is no impurity. So long as only cold affects a tooth with capped nerve it is healthy. If heat is painful, there is inflammation—the nerve is dead or dying.

*Dr. H. J. McKellops*, of St. Louis, said that the opening necessary depended upon the tooth and the patient in each individual case. In the case of a young patient, with a large cavity running into the cusp, if not well excavated, the tooth will turn black. Gold must be adapted to the walls of the cavity to save the tooth. If the top is left intact you cannot see what to do. In dead teeth you have to cut out well and see what you do. Spoke of the nodule rolled around in the cavity and finally impacted in the filling (as described by Dr. Morrison). Said that such a substance was diseased matter and should be removed; that neuralgia in apparently sound teeth arose from calcified pulps. Asked Dr. Morrison why he left that foriegn substance in the cavity—had never heard of such a thing before—would treat through the foramen and be successful, but if there was a blind abscess, trouble was inevitable. Cited several very stubborn cases in which final success was attained. One case, a young lady with abscess over superior central and lateral incisor. Had been treated by a New York dentist who made a specialty of alveolar abscess; had been going on four years—lady in poor health, wasting away—diagnosed filling projecting through the apex, with necrosed bone to the base of the nose; removed fillings of gutta percha and cotton from the root; opened up the side of the teeth; removed sixteen pieces of bone. There was no remedy but to remove the teeth; treated every three or four days, for five or six days. (Exhibited the teeth.) Said: My friend from New York does not practice what he preaches. I would not dare to cut out as he does, but I cut enough to see what I am doing. As to filling roots with gold, every one knows what I think, I have preached till I am ashamed to say more. Dr. Clark, of New Orleans, taught me his method. There are cases where gold can not be got in; you must have something you can force up—that is gutta percha. No matter how skilled a man may be, he can't fill a blind cavity with gold, but you can inject gutta-percha, and drive it up, and do no harm, where gold would go through and cause excruciating pain." Dr. McKellops then described a recent case where the cheek was swollen, and feeling like dough, the abscess approaching an outside opening. Extracted the tooth with the abscess intact—a round ball on the apex—the root had been filled with gold which passed through the apex, with an amalgam crown. To handle gutta percha for root filling, imbed a stiff broach in unslaked lime and heat it red hot, making it so stiff as to require careful handling, wrap with threads of fine cotton; with this you can force gutta percha where you cannot get gold. (Exhibited another tooth with cotton forced through the apex.) If a man takes pains, he can't be in a hurry; he can't do first-class work in a hurry, he makes himself hard work in the future. Dr. Shepherd uses waxed tape to separate the bicuspids—takes a week and has no soreness—you can get plenty of room and see what you are doing. I don't pretend to succeed in all operations. I fail, and others fail. We don't always know of our failures; they are tired of us and don't come back. I am ready to give any man any amount of money that can show me how to do these things in the best manner. I have seen the best gold work fail, and I have seen amalgam fail. I have seen failures with people who take the most exquisite care of their teeth, spending hours on them daily, people with whom money was no object. Gold work failed with them, and they came back; I put in amalgam and that failed. I put in oxy-phosphate and renew it every year. It is all I can do. If any man can tell me how to treat such cases, I want to learn. Some one in the *Southern Dental Journal* recommends oxy-phosphate where patients are willing to have it renewed every year—it is the nearest approach to success possible in some cases. Gave the following illustration: A young lady with the superior left anterior bicuspid, with the palatine cusp broken half-way; the tooth very frail; the outside wall of enamel cracked, but the nerve alive; the mother standing by, anxious that the tooth should be saved, yet exclaiming, stop! stop! in her fear that the tooth would be broken off. Three times in seven years I have filled that tooth with oxyphosphates, and it is in good condition to-day. Such work requires time and patience, but it will secure good results. Of course we cannot expect perfection with such teeth, and patients must be made to understand this, and expect and agree to have the work renewed. Cited another case, that of an inferior right posterior bicuspid; cavity on the labial surface extending so very far down that the gum had to be pressed away, and dentine very sensitive; had entire success in producing local anesthesia with the following formula:

| | |
|---|---|
| Muriate cocaine, gr. - - - - - - - | 1½ |
| Spr. Alcohol, dr. - - - - - - - - | 1 |
| Chloroform, dr. - - - - - - - - - | 1 |

Blistered the mouth but saved the tooth. In filling root canals use gutta percha in chloroform. Use No. 10 Swiss broaches, they are highly tempered and must have the temper thoroughly drawn before using.

*Question*—Will not the chloroform evaporate and cause shrinkage of the mass in the canals?

*Dr. McKellops*—The evaporation takes place during manipulation, before it goes into the tooth.

*Dr. C. W. Spalding*—I don't propose to offer arguments against the practice of others. I don't presume a man would advocate a measure if he had not been successful with it; but no one has claimed to fill root canals with gold only, and have the work perfect. No such claim has been made in the papers read, nor in the discussions.

*Dr. McKellops*—Did you not say "close the apex with gold."

*Dr. Spalding*—It does not much matter what material is used, provided you reach the apex; but nobody advocates filling canals with gold wire only, leaving interstices. We use plastic, or a solution of gutta percha, but at the same time to make sure that the apex is closed, we take the measure of the depth of the canal and cut off the right length; then having filled the canal with the plastic material, insert the gold wire in the plastic mass, and you drive it into all the interstices, and seal the apex. Much has been said against the process, but it gives the nearest approximation to absolute certainty.

*Dr. H. A. Smith*, Cincinnati—Would emphasize what Dr. Harlan said; must exclude foreign substances, and agree with Dr. Parmly Brown to make haste slowly. Take a tooth in a pathological condition; animal matter must putrify; every time we apply antiseptics we make progress towards cure, but must maintain that progress by isolation. What is most needed now, is the means of protecting a tooth from the invasion of foreign elements while under treatment.

*Dr. Taft*—Most of the remarks made in the discussion seem to be based on the supposition that dentine is uniform. Must note whether tooth is living or devitalized; whether a tooth is young or matured, difficult to treat devitalized teeth in young persons, that contain more organic matter. If, as Dr. Morgan says, there is decomposition in the tubuli, it must be much worse in young teeth, there is more liability to periostial irritation, from emanations passing through this less dense dentine. All these conditions should be recognized and considered. Some adult teeth also are less dense than others; some are very soft; this depends on various causes. If the tooth is firm and dense, we can devitalize and fill immediately; we can perform operations that would not be tolerated at all in soft teeth. There are some teeth with which we can do almost anything; others that are difficult to save; others in which success is impossible. The contents of the tubuli do not always undergo prompt decomposition. The evaporation of water from the tissues often prevents decomposition. Different constitutions act differently under irritants. Some take up poisons and eliminate them without disturbance. In others the slightest irritant will produce violent disturbance. We must discriminate between youth, middle age and old age; between health and debility; be guided by conditions. We must study the histological character of each case. The electric light is an invaluable aid in distinguishing the conditions of the teeth. The condition of the dentine also modifies the amount of cutting away; dense teeth have solid walls. In soft teeth remove as little as possible; in dead teeth there is a retrograde metamorphosis.

*Dr. Morgan*—I take exception to the position of Dr. Harlan, regarding the removal of watery portions of contents of tubuli. It will be renewed from the periosteum. In proportion as you apply antiseptics you prevent discoloration. So much for the organic matters. I will tell you how you can fill a root and know the apex is sealed. If you drill all the way through with a cone-shaped burr, you have a cone-shaped opening; the bur is the model for the filling; make a cylinder cone of gold; carry it up and drive it home firmly, and you have it sealed.

You can plug a crooked root with gold and know the result. With the first soreness of the periosteum, by watching closely you can prevent an abscess by applying aqua ammonia as a counter-irritant, and putting the system in condition to resist inflammation. When an abscess has been treated the bony tissues are broken down and there is a filling up of abnormal connective tissue. When the cause of irritation is removed nature strives to renovate; the absorbents become active; new tissue is formed, but it is not the original tissue; it is eschar tissue.

*Dr. J. R. Patrick*, Ills.—Roots have all shapes; they are straight or curved, club-shaped or twisted, but as a rule the internal wall follows the external outline, but you can not be positive even of this. When roots are filled with wire, it is liable to perforate the apex. I have seen a tooth with gold wire projecting one-half inch beyond the foramen. It is true, it had been worn for years without trouble. With regard to fistulas, I have found the treatment easy in the lower teeth; by the law of gravitation they drain naturally through the fistulous opening, and you can fill at once. But it is different with the upper teeth; they drain through the canal, and if you risk filling at once you have to rely upon absorption.

*Dr. Wm. N. Morrison*, St. Louis—The discussion has been too long already, but I wish to reply to one or two points. First, as to what Dr. Patrick says about wire passing through the apex, I doubt if he will ever find a filling of root projecting half an inch. If the wire goes too far on trial, it is very easy to substitute a larger size until you find one that does not pass. I will gladly illustrate my method in a clinic on Friday. Select your most difficult cases, either live teeth or dry teeth. If dry, invest them in plaster, so that I can not know the shape of the roots. I would prefer teeth in the mouth. If in plaster, I will ask you to break them open, and judge of the work in the canals. I will be glad to put it to this test. (Applause.)

*Dr. Moore*, S. C.—I must challenge one little point in Dr. Morrison's system. He may be able to withdraw all the pulp tissues through an aperture of one-sixteenth of an inch in diameter, but it would be possible to but very few of us. I would prefer to sacrifice a little more of the dentine of a devitalized tooth. Discussion closed.

The following new members were added to the roll, having passed favorably the Executive Committee.

Dr. S. H. Bartholomew, Vicksburg, Miss.

Dr. E. B. Robbins, Vickburg, Miss.

Dr. W. I. Barton, Paris, Texas.

Dr. L. A. Thurber, New Orleans.

Dr. W. L. Smith.

Dr. G. A. Colomb, St. James, La.

Dr. M. W. Williams (Ky.) read a paper describing a new artificial crown, illustrated by a large chart, showing the different portions of the attachment. The following is a brief description of the device, omitting details. Two hollow truncated cones of soft platinum are inserted, one in the root canal, and one in the artificial crown.

The foramen is first closed with the point of a lead needle, and the root canal filled with cement; one of the hollow cones is inserted, and being open at the end, fills with cement. A short, broad steel pin, split at both ends, passes from root to crown with a steel wedge in each slit. An approximal point being made and amalgam spread thinly over the end of the root, the crown is driven home, and bitten down into perfect articulation, the wedges expand the ends of the pin so as to fill the inside of the caps, drawing crown and root together, forcing out all surplus material and securing a perfect joint, the wedging principle preventing any possibility of opening or separating, and apparently rendering the attachment as durable as the root itself. The device was received favorably, commending itself in being self-articulating, by its strength and simplicity, and by the ease and rapidity with which it can be attached in one short sitting.

After a few questions and explanations the paper was referred to the Committee on Publication, and the subject of operative dentistry passed.

The subject of Pathology and Therapeutics was now called for. The paper of Dr. A. O. Rawls on pyorrhea alveolaris, incorporated in his annual address and read on Tuesday, was taken up for discussion.

*Dr. J. R. Walker*—Said that pyorrhea alveolaris had been the *bete noir* of the profession for long years. Regretted the absence of Dr. Riggs, who is entitled to have the disease called by his name, as his researches have at last given us a clue to the only successful method of treatment of what was so long considered an incurable disease. Facts in obtaining best results by surgical treatment alone—uses local applications as adjunct in restoring normal condition of soft tissues after surgical operation has removed the local irritants.

Have found the following very beneficial in inducing healthy granulation, and the filling up of pockets:

| | |
|---|---|
| Carbolic acid - - - - | 1 |
| Tincture iodine - - - | 1 |
| Glycerine - - - - - - | 10 |

When well mixed add 5 parts Laboraque's solution. It becomes perfectly colorless, and more volatile than water. Then add a few crystals chloride zinc; it coagulates and is very astringent.

*Dr. Reese,* N. C.--The most important point is to study the cause. Pyorrhea is rather a symptom of disease than a disease *sui generis.* From my observations it is caused by the use of alcoholic stimulants—not immediately but in the course of years. The use of alcohol increases the uric acid in the system, urea having also a strong affinity for sodium. The deposits never occur until after the destruction of the peridental membrane. Have had the deposits analyzed: it is pure uric acid. The deposits occur farthest from the heart's action, and in parts of low vitality. It is confined to men; women never have it until after the cessation of menstruation. Negroes never have it. Remedies which decrease uric acid in the system, as atropine, digitalis, acetate potash, etc., are the remedies for pyorrhea.

*Dr. W. H. Morgan.*--In accordance with the medical axiom, "remove the cause and the disease will cease," but I had never heard that the calculi of pyorrhea were uric acid. It is most commendable to investigate in this direction. Have not had it analyzed myself. As to the other statements; in my town there are regiments of negroes with genuine pyorrhea, and women of all ages have it as commonly as men. Have treated at the same time a mother with a suckling child, and her daughter, a girl of fifteen years old. Have treated it in three generations of one family, from the grandfather down, and know that no stimulants were used in that family. When the health is broken down from this disease, as often happens, general as well as local treatment is required. If it is of constitutional origin it requires constitutional treatment. It is often miscalled scurvy because of some points of resemblance.

*Dr. Taft*—There exists a great variety of opinion as to the origin or causes of this affection. We are told of systemic conditions, but we are not informed what they are. There is no doubt an enfeebled condition, in many instances, which promotes it. This may occur from defective elimination. When the teeth are not properly used, there is lack of nutrition, and the gums are very susceptible to irritants. Systemic conditions may arise from imbibition of pus, in pockets whence it cannot escape. If the tissues are not nourished, or if there is lack of elimination, adverse systemic conditions will result—until this is rectified, local treatment will be of little or no avail—the system must be toned up to better nutrition and better elimination. We must study principles rather than mere remedies. It is a great fallacy to rely on any one agent. We must do the best we can, taking into condsideration all the conditions of each case.

*Dr. J. A. Robinson*, Michigan.—I have given the profession a remedy which I have called Robinson's Remedy. It may be called carbolized potash. I remove thoroughly all deposits, using chisels, cutting toward the necks. Equal quantities of crystals of carbolic acid, and crystals of caustic potash, (with a very little water unless the crystals have deliquesced) will form a creamy paste, which will liquify. Make small ropes of cotton the size of floss silk, cut off in lengths sufficient to reach around one tooth at a time, dip the pieces in a bottle and lay on a napkin to absorb the surplus; the gums will slough if too much is used; place in the pocket or under free margin around the tooth, and leave on one tooth only while cleansing another, the next tooth; a second treatment is seldom necessary, and have never made a third application. When there is much pus, the rope comes away brown, or even black. It would seem to be a specific remedy; produces a change in twenty-four hours that would scarcely seem possible. (Described a number of very extreme cases, with marvelous results.)

*Dr. Walker*—I hope Dr. Robinson's remedy will do all that he claims for it. As different cases require different remedies, one succeeding where another fails, I have given a remedy which I have found very efficacious. Dark blood changes from blue to red, and looks lively and healthy, and pockets fill up rapidly.

The hour for adjournment having arrived, further discussion was deferred until the next session. The committee on Memorial Resolutions announced the following resolutions relative to the death of Dr. S. M. Prothro, of Chattanooga, which were adopted by a rising vote:

WHEREAS, it has pleased Almighy God, who doeth all things well, to call from our midst Dr. S. M. Prothro; therefore,

*Resolved*, That we bow submissively to the divine will, and that we as a profession have lost a worthy member, always upright in thought, just in his dealings to all, capable and efficient, bearing with patience and fortitude the ills of life.

*Resolved further*, That we as an association extend to the bereaved and stricken family and friends our heartfel sympathy in their hour of affliction.

G. S. CHISHOLM,
W. C. WARDLAW,
W. H. RICHARDS,
Committee.

Dr. Morgan paid an eloquent tribute to the memory of Dr. Prothro.

Dr. Prothro was a native of South Carolina, and was fifty years old at the time of his death. Graduated when but eighteen years old, and received his medical diploma before he was twenty-one. He took up the practice of dentistry after 16 years of medical practice. He was a man of natural sprightliness, gifted in conversation, and ready with the pen; an elegant gentleman, cultivated, refined, and high-toned; remarkable for the purity and simplicity of his character. His example is one worthy of imitation. His memory should be held in affectionate remembrance.

Adjourned to meet at 7:30 P.M.

SECOND DAY—NIGHT SESSION.

Called to order at 7:30 P.M. The President, Dr. A. O. Rawls, in the chair.

The subject of Pathology and Therapeutics resumed, by the continued discussion of the paper on Pyorrhea Alveolaris.

*Dr. Harlan*—Said that opportunities of studying the sailors of the high seas were comparatively rare for practitioners of dentistry in inland cities, and that his practice did not lie much among Irish laborers, but he did not think the deprivation of vegetable food or the use of salt meat had any significance in connection with pyorrhea alveolaris; that their diseases of the teeth and gums were more probably due to their utter lack of all ideas of cleanliness. They might have salivary calculus, but not necessarily pyorrhea. Such theories as these were condemned. The descendants of the users of salt meat do not inherit pyorrhea. The disease of the gums after ptyalism does not resemble pyorrhea. The mercurial diathesis may be transmitted, but not as acute ptyalism. So much for Dr. Rawl's theories of *causes*. He well said that escharotics should not be used, but do not agree that germicides or antiseptics exert no beneficial influence when properly applied. Dr. Rawls stated that the septum might melt away but the gum left on the original line. It strikes me that that does not obtain.

In mouth-breathers, the palatal aspect of roots of incisors is affected. Salivary calculus has nothing to do *per se* with the inception of pyorrhea. After the removal of salivary deposits, the peridental membrane often remains well attached, so that the probe will not pass. Pyorrhea deposits go to the apex, but not continuous; there is no particular point of large accumulation of serumal calculus, but rather in little islands. The pockets are more frequent on labial and buccal aspects than between the teeth. When on mesial or distal surface, it is usually where the adjoining tooth is missing. The thorough removal of all deposits is absolutely indispensable; the edges of bone must be broken down, cut away, burred out, scraped out. A frequent cause of failure is the failure to remove the debris. This cannot be thoroughly done merely with a syringe and water. It will often be found necessary to make a transverse slit in the gum, to make certain, thorough work. If made purpendicular it must be extensive to prevent pucker of the gum in festoons; thorough work on mesial and distal surfaces only possible by a transverse cut through the gum. On the labial surface it is easy to push away the gum. Exclude saliva and inject peroxide hydrogen, full strength as it comes to us. It will make more perfect work them would otherwise be possible. The blood clot which in other positions furnishes nature's protection is not transformed tissue. What we most need is something that will destroy bacteria. The fungus of pyorrhea is not yet satisfactorily classified. That true pyorrhea is infectious I have demonstrated in one mouth, when I purposely introduced pus from pyorrhea beneath a healthy gum; you may call the result satisfactory or unsatisfactory, but the tooth was lost. The moral is that our instruments must be cleansed in preparations that will destroy spores, if we would not propagate the disease. There is no advantage in over treatment. Every three or four days is enough, with intervals of rest in which we allow nature to work.

*Dr. J. P. Knapp*—The disease under discussion is not a new disease. It is not Rigg's disease more than anybody else's; for it is older than Riggs himself. His treatment, however, is good, in the thorough removal of all deposits and debris. If it was caused by tartar, all deposits of tartar would be accompanied by pyorrhea, and there would be no pyorrhea without tartar, neither of which is true. Failure in thorough removal of all deposits, debris and dead bone, resulting either from lack of boldness in the operator, or from the flinching of the patient, is the usual cause of failure to cure; use chloride of zinc in pockets, place a few granules on a piece of glass with just enough water to dissolve; apply with instrument of wood; after three or four minutes, wash out, and follow with tinc. iodine. It will be slightly uncomfortable and cause temporary discoloration, but will have a beneficial effect. It will harden up the gums and prevent bleeding. Cannot agree with Dr. Atkinson that the alveoli will be reproduced after the disease is arrested. It may not be impossible, but it must be the work of years.

*Dr. J. R. Walker*—If any one man in our profession deserves to have his name perpetuated it is Dr. Riggs. Thorough surgical operations; the adoption of the surgeon's axiom, "cut beyond the dead line;" the thorough removal of everything down to sound bone is the true theory. Dr. Riggs was the first to go that far; to teach us, and make us appreciate the necessity of daring to do this. Too much credit cannot be given him, for to him we owe the possibilities of cure, under the modern system of treatment which is the result of his investigation.

*Dr. A. O. Rawls* (calling Dr. Wright to the chair)—I find that I am still severely misconstrued. My object in reading the paper was to put myself right before the Association. Dr. Knapp and Dr. Harlan both attribute to me statements entirely contrary to my views. I have contended that when the process is broken down there is no possibility of absolute cure. Before the process has been reached there is a simple inflammatory action that may be checked, and the progress of the disease arrested for a time, but the conditions are still present.

I have never found a case that was not due to the use of mercury or of salt. The victims of malarial poison have an abnormal craving for salt. It is the only diseased condition that creates this craving. You also know the prevalence of pyorrhea in malarial districts. See also the condition of sailors and Irish laborers, who live almost exclusively on salt meats. I have made it my business to seek out these two classes of people, and invariably find these conditions present. My theory may be wrong, but the coincidence exists. It has been said that "reproduction can take place. Syringe the pockets with this and that and allow the tissues to build up."

Will live tissues grow over dead bone when the process has been denuded of the membrane? There is no possibility of reproduction when the connection between the tissues and the bone is broken. In soft tissues the function of nutrition is resumed. I do not say that mercury will always produce this disease, but I do say that in nearly every case mercury will produce conditions that will render possible this peculiar disease.

It is touching on dangerous ground to deny that it may be produced by fungi or bacteria, but it is certain that they follow the destruction of tissue—the resultants, not the starting point. Antiseptics or escharotics to destroy these animals are outside of the question; our object is the restoration of tissues; not killing germs; that does not cure disease. We must remove the sources of irritation; remove the dead bone and debris. It is a simple process, but it is all we can do, with the after protection of the parts.

*Dr. Taft*—What does Dr. Rawls consider hereditary mercurialization? If a person has had this special mercurial impress is its transmission from parent to offspring probable or possible?

*Dr. Rawls*—It is as transmissible as any other condition of tissue. Conditions are transmittible. Every molecule is marked by its surroundings, as seen in the curl of the hair, or the color of the eyes. What is heredity? It is the transmission of the peculiarities of the parent to the child. Diet, surroundings, environment cause peculiarities which are transmittable in the tissues of the gums the same as elsewhere. It is in this way that the sins of the parents are visited upon the children unto the third and fourth generation. The tissues of mercurialized persons are by no means as strong and firm as others. They are readily broken down. This condition is the same in transmitted tissues as when acquired.

*Dr. Taft*—How permanent is this effect of mercury? Is it never eradicated?

*Dr. Rawls*—I do not say that. Changes are taking place all the time in man, in his relations and environment. All these have their bearings on the laws of heredity.

*Dr. Taft*—When tissues have been affected by an agent, does the effect continue after the agent is expelled?

*Dr. Rawls*—The tissues can change, and do change; but an impression has been made which renders the tissues susceptible to irritants. The impression is made on each molecule; a change is made in the constitution of the molecules; the general impress is on the molecules, and it is transmittable; not the agent itself as mercury for instance, but the impress made is permanent. It is like making a new chemical compound by the addition of another element.

*Dr. Taft*—The transmission of types inherent in the constitution of physical peculiarities, as in hair or eyes, is admitted by all; but that such accidents as the exhibition of medicines with evanescent effects varying with the power of the system to rally, that an impress so temporary as the effect of mercury, is transmittable, appears to me doubtful, to say the least. There are some things that are never eradicated; that are so permanent as to be ineradicable, as syphilis for instance, which may be, and doubtless is transmittable, but I doubt the transmission of the mercurial influence. The system may be weakened and broken down, and children consequently not robust, but the transmission of short-lived accidents appears doubtful to me. It is dangerous ground to accept such theories.

*Dr. Morgan*—I want to ask if all radical changes made in the system, and continued, are transmittable?

*Dr. Rawls*—If not, you could not live a moment. What makes you what you are? Not subtle influences that can neither be felt, seen nor handled, but the food you eat, the air you breathe; they make you what you are, and their effects upon your system and your tissues are transmittable.

*Dr. Morgan*—Then how is it that we who are lame all our lives have athletic sons? An eye knocked out is not transmitted. Reproduced alveolus is never in the original form. It will be largely increased in bulk and strength. I once saw a man who carried a large piece of the outer plate of his skull in his hand. New process was being formed, with healthy tissues. The periosteum is reproduced, and granulation takes place.

*Dr. Robinson*—Dr. Morgan has told me that the rheumatism which caused his lameness was inherited, and we do not know that he will not transmit it.

*Dr. Cushing* (to Dr. Rawls).—What are the physical signs of these transmitted conditions?

*Dr. Rawls*—Pyorrhea alveolaris presents different aspects when transmitted or when acquired. In transmission the tissues are softer and less healthy; there is not the same integrity; they break down more rapidly. The lameness of Dr. Morgan not being inherited is no evidence that the conditions which made it possible were not, or that he may not transmit to future generations, though not to his son. The world did not grow in a year. The transmission is in general conditions, not in local forms; in types, not the accidental impress from an abnormal element.

*Dr. Walker*—The fact is that the more we search for and understand causes, the better we can treat the effects or results. The observations of Dr. Rawls lead him to look upon salt and mercury as factors of causation. The deposit of calculus is evidence of misdirection of elements.

*Dr. Fountain*, Texas—In the Brazos bottoms, pyorrhea is very prevalent; perhaps from malaria, perhaps from mercury. Most of my patients say they have been salivated.

*Dr. Patrick*—There is no doubt that the physical impress is transmitted. Proofs are very strong. A concatenation of circumstances bringing about certain results, account for those results. To illustrate: In Sedalia, Mo., a gander, in foraging for food along the railroad, got under the wheels and had his wings broken. The next spring a number of goslings were hatched with broken wings. A photograph of the group was sent to Chas. Darwin. At his request, the gander was placed the next spring in another flock, six miles away from the railroad. There were no more broken winged goslings. Circumstances and surroundings were all changed.

We will probably never know *why* one family for several generations have the two first fingers missing, even first cousins sharing the same peculiarity. There is no explaining it. It is so; that is all. The fact is established, but why or how we don't know. We find this illustrated in the teeth as in the other organs.

*Dr. J. A. Robinson*—I can cite one family when for four generations, to my knowledge the grandfather, father, daughter, and her little child, have no upper lateral incisors.

*Dr. Teague* (S. C.)—Cited the cases of two well known families in South Carolina who have been edentulous for generations. Also another family known for three generations as inheriting a hideous deformity of double hare-lip.

The discussion was closed.

Dr. J. A. Robinson, of Jackson, Michigan, then read a paper entitled, "How to Increase Your Usefulness."

This paper was replete with sound advice addressed especially to the younger members of the profession, embodying the wisdom gained by the experience of a veteran in the service, "Father Jerry," as he is affectionately called by all who know him well, having passed the limit of three-score years and ten. He began by quoting the well-known saying of Daniel Webster, "There is always room at the top," regretting, however, that the most competent dentist, he who has reached the top of the ladder in professional ability and professional estimation, was apparently little appreciated by the community in which he lived, judging by his position on the financial ladder. Push without ability will accomplish nothing, and ability without push will not remedy this state of things. Individually, we only catch glimpses of the truth, like the gray before the dawn. It required a Franklin, then a Morse, and then a Field to perfect the telegraph. The beginning of a new truth, when first seen, seems to threaten to sweep us beyond the ancient shores we thought secure. In the great inundation of the light of to-day, it is hard to distinguish what is solid ground. Our improvements grow with our affections. Being just to himself and to others, the affections of a just man are wounded by failure; he must abandon the means and the methods which occasioned the failure; hence his improvement. To be successful you must do the things that other men do not do, and to do this requires study and effort. The first means to this end is to read all the journals, that you may know what others have done, and what is left undone, for you to attempt. If the same article is published in three or four journals, read it three or four times. It would not be published so often if it was not worth it. As special fields of study, take diseased teeth and gums. This is a broad but comparatively new and uncultivated field to the average dentist. The same is true of artifical crowns, which are rarely attempted except in large cities; correcting irregularities is another important branch that is comparatively ignored by the average practitioner, while one of the most delightful branches of our art. It broadens the field and affords relief from the ordinary routine of chair-work. It develops high art, and elevates the dentist in the eyes of the community. It reveals new beauties in the human face divine, correcting deformities that would be remedied only by the sacrifice of good teeth for the substitution of artificial ones. This is a field far more practically useful than the embryonic, microscopic, and other studies upon which the student spends so much time. The cultivation of the fraternal feeling in the profession is one of the most dignified means of securing increased patronage. Let the people see that we have confidence in each other and they will have more confidence in us. It is the rule of society everywhere. Secrecy and acquisitiveness are twin sisters: together they dwarf the mind. Hidden somewhere, like diamonds lying in the depths of the ocean, of untold value when found, are the materials capable of being so combined as to give us that ideal filling which shall truly restore the mutilated tooth to its original form and usefulness. If my premonitions are true, this will yet be found. Who among you will be the finder? One of the first qualifications for success is to have the courage of your convictions, and personal force enough to impress your patrons with the principles and truths you wish to instill; make them feel your enthusiam as you feel it yourself. We may love the past, but we must look to the future. There is an immense realm of possibilities yet unexplored. It once seemed that Mozart and Beethoven had exhausted all the possibilities of musical sounds, but Mendlesohn and Wagner revealed new harmonies undreamed of by the old masters. Sometimes we think we love the truth when we only love our own ideas of what is true. We are here to fulfill the end for which we were created; it was not in the scheme of Providence that man should be sick, or that we should lose our teeth. The main business of the dentist is to save the natural teeth, by the best means, and with the best material within his reach. Our success will depend on our ability to make our work permanent. Artificial dentures only serve to make things bearable till we go down to the grave. A man is not fit to be a dentist unless he acts on this principle. We must deal with principles above mere mechanism. The truths of science, the laws of nature, the law of justice and right, of truth and love, must be unitized in a profession to make it felt in the world. Cleanliness is the first law of nature, God Almighty is constantly washing all nature with his dews and rains. Man also must be obedient to this law. Filth, fermenting and putrifying, is the cause of decay. Tooth-extraction is not a part of our business. It is not a part of our mission to assist nature in working out the law of evolution by which the human race may become toothless. Edmund Burke said, "Where bad men combine, good men should combine." We should band ourselves together against the bands of charlatans. And all this we must not only believe ourselves, but we must make the world believe it. Belief lies back of conduct, as force lies back of motion. It is only what is fully believed that is acted up to. And so, the last but not the least means to increase your business will be modest, dignified advertising; direct and indirect; by card or by newspaper; by the omnipotent power of talk. By the free use printer's ink we elevate and enlighten the world. An important matter for consideration is the lack of sufficient compensation for our labor. The world is full of equivalents; man is ever unwilling to give more than he receives.

The demand for cheap dentistry is founded in popular ignorance of the value of the teeth. Here the press is the most efficient means of spreading abroad the truth. There is less general information regarding dentistry and the teeth than on other subjects. Here we have the key to the situation. We must make the people a part of ourselves, if we expect their patronage. There are trades unions to protect the working people; there are conventions to lift the masses; there are expositions to enable the people to behold the world at a glance. We owe this to ourselves, and to a long-suffering humanity; to the rising generation. We owe it to the progress of the age; we owe it to a profession that has outstripped all others in rapid advancement. Consultations in medicine are paid for whether treatment follows or not; the lawyer has his retaining fee; the dentist only is expected to give his time for nothing—to spend more time in gratuitous examinations than he does in actual labor. Information regarding the value and the care of the teeth should be embodied in school-books from the primary to the graduating class. Light should be shed on this subject by the daily or the weekly newspapers.

The man who will thus stand by his convictions has also the right to attach his name to such information. And when we have done this, we shall have our reward.

I have followed my profession for forty-nine years, and I have always tried to do well, but I am free to confess that I have never felt the responsibility of my calling as I do to-day. Rubens said, when he was ninety-three years old that he had just begun to improve. We will increase our business just in proportion as we know what to do with tooth sinners to save them, and what to say to tooth saviours to make them faithful to their high calling.

(This is but a brief abstract of a paper which was also full of practical suggestions and information as to the latest means, methods and materials in operative dentistry.)

*The Chair*—This most excellent and practical paper is now open for discussion.

*Dr. Taft*—It is very late. I move that the discussion of this paper be postponed until the next session. Seconded and carried.

Adjourned to meet at 9 A. M., to proceed to the grounds of the exposition, for the celebration of Dentist's Day, Thursday, April 2d.

Friday set apart for Clinics and the exhibition and explanation of Appliances.

Next regular session Friday, 7 P. M.

